# Jiawei Xiaoyao San in treatment of anxiety disorder and anxiety: A review

**DOI:** 10.1016/j.chmed.2022.12.007

**Published:** 2023-03-15

**Authors:** Jiaqi Xie, Dandan Xu, Can Wang, Jianmei Huang

**Affiliations:** School of Chinese Materia Medica, Beijing University of Chinese Medicine, Beijing 100029, China

**Keywords:** anxiolytic activity, anxiety disorder, Jiawei Xiaoyao San

## Abstract

Jiawei Xiaoyao San (JWXYS) has shown excellent clinical efficacy in anxiety disorder, but has not yet attracted widespread attention. The animal experiments, clinical trials and mechanism studies of JWXYS were reviewed in this article, which may provide a reference for developing new anxiolytic drugs based on this prescription. The literature was searched in PubMed and CNKI and the documents written in English or with English abstracts were selected. JWXYS could reduce the anxiety symptoms of patients alone and reduce the adverse reactions when it is used in combination with other drugs in the clinic. In preclinical studies, JWXYS also showed therapeutic effects in reducing anxiety-like behavior. The mechanisms may include improving the hypothalamic–pituitaryadrenal (HPA) axis and hormone disorders, increasing neurotransmitter content, neurogenesis, and regulating the synthesis of related enzymes. This article shows that JWXYS could effectively treat anxiety disorders by regulating the central nervous system. In the future, with the participation of more researchers, it is expected to develop innovative drugs for the treatment of anxiety disorders based on JWXYS.

## 1 Introduction

Anxiety disorder is a common mental illness with persistent fear or anxiety (typically lasting six months or more) and related behavioral disturbances (American Psychiatric Association, 2015). For a long time in the past, the incidence of anxiety disorder could not be accurately counted until there is a clear diagnostic criterion. In recent years, demographic data in the UK shows that 3% of the population were eligible to be diagnosed with anxiety disorder, but only 8% of those identified were diagnosed with anxiety disorder finally and received relevant treatment (Slee et al., 2019). In particular, anxiety disorders are considered to be one of the most common psychiatric disorders, affecting 15%−20% of young people (Kessler et al., 2007, Merikangas et al., 2010). Corresponding to anxiety disorder, anxiety is a relatively widespread type of human mental illness, that has emotions of dread, apprehension, and impending disaster but not disabling as anxiety disorders (Bandelow, Michaelis, & Wedekind, 2017). Anxiety can be adaptive as it is a response to danger or potential threats in the environment (Kalin, 2020). The statistical results show that the global overall prevalence of major depression is 4.7% (Ferrari et al., 2013), and about 85% of the patients also have symptoms of anxiety (Tiller, 2013). It is important to note that both anxiety disorder and anxiety can have bad consequences (Celano et al., 2015, Rotvig et al., 2022). It is urgent to find a suitable therapeutic schedule for anxiety disorder and anxiety.

At present, the treatment of anxiety disorder is still based on drug intervention and there are a variety of drugs to choose from the clinic. The efficacy and tolerance of drugs are different, which made the drug regimen of different patients has certain differences. The first-line medication is chemical drugs, but the price of drugs is generally high, which is a great expense for many patients (Konnopka & König, 2020). At present, the main purpose of clinical medicine is to relieve symptoms. A total of 50% improvement on the Hamilton Anxiety Scale (HAMA) or being able to live normally after stopping the drugs rather than completely curing the situation are commonly used as the endpoint indicators (Baldwin et al., 2011, Noma et al., 2019). What's more worrying is that these patients still tend to show symptoms of chronic anxiety in the future (Kasteenpohja et al., 2018, Roy-byrne & Cowley, 1994). According to the survey, the lifetime prevalence of generalized anxiety disorder worldwide is about 3.7% (Roy-Byrne, Craske, & Stein, 2006, Ruscio et al., 2017). Therefore, it is necessary to find a cheap and effective anxiolytic drug.

**Fig. 1:**
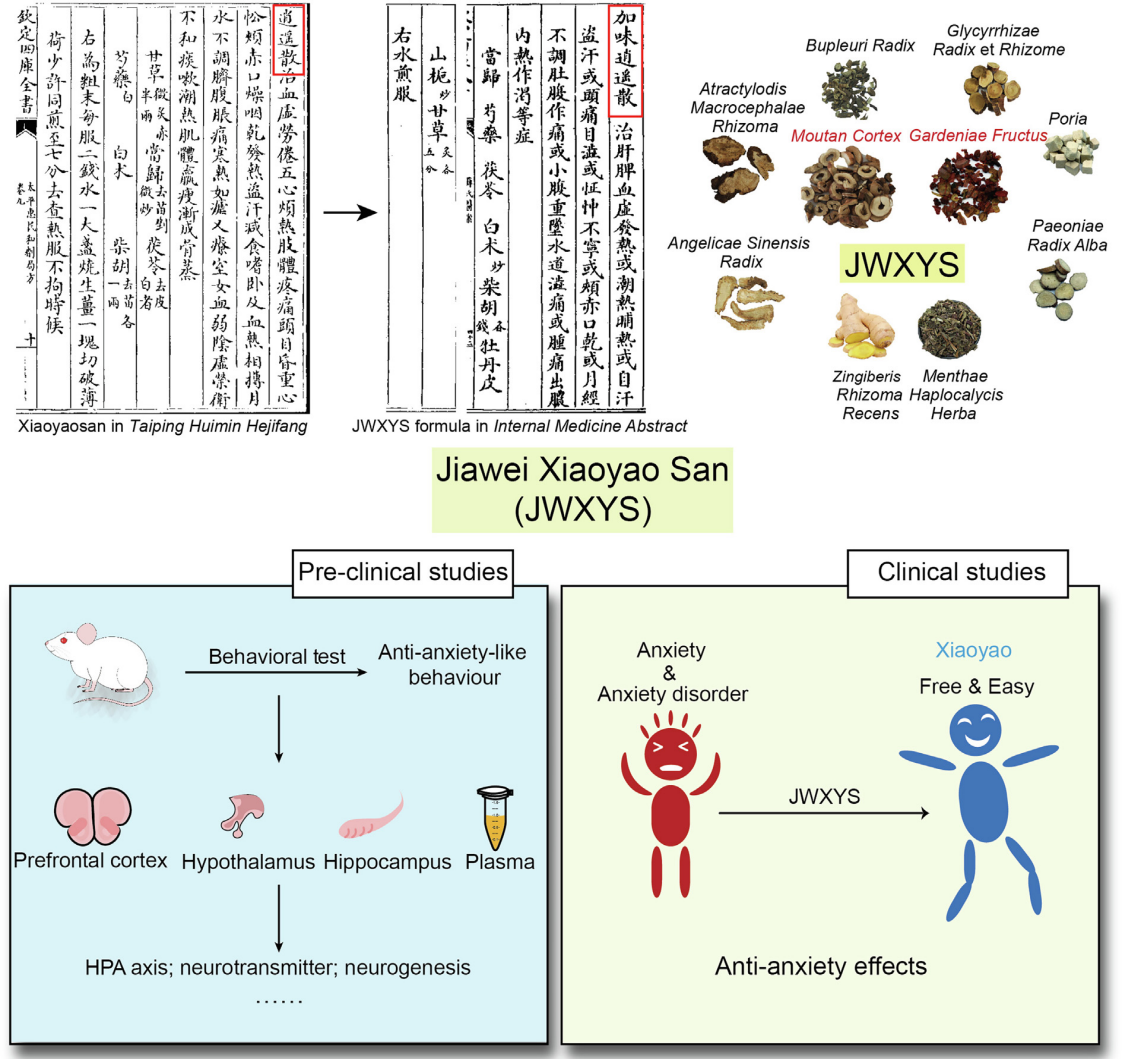
Origin, composition and studies of Jiawei Xiaoyao San (JWXYS). Xiaoyaosan was recorded in the world's first drug standard *Taiping Huimin Hejifang*. JWXYS was recorded in the *Internal Medicine Abstract* published in 1529 CE, which is composed of Xiaoyaosan and two other Chinese herbal medicines. JWXYS is composed of 10 Chinese herbal medicines. JWXYS has shown good anxiolytic effects both in pre-clinical and clinical studies.

Traditional Chinese medicine (TCM) may show unique advantages in the treatment of such psychiatric disorders. Jiawei Xiaoyao San (JWXYS) is a traditional Chinese medicine preparation and its composition has been derived from the *Internal Medicine Abstract* published in 1529 CE (Fig. 1). It is derived from the drug ‘Xiaoyao San’ in the world's first drug standard *Taiping Huimin Hejifang*, two Chinese herbal medicines were added on this basis. JWXYS is composed of 10 Chinese herbal medicines: *Angelicae Sinensis Radix* (Danggui in Chinese), *Paeoniae Radix Alba* (Baishao in Chinese), *Poria* (Fuling in Chinese), *Atractylodis Macrocephalae Rhizoma* (Baizhu in Chinese), *Bupleuri Radix* (Chaihu in Chinese), *Moutan Cortex* (Mudanpi in Chinese), *Gardeniae Fructus* (Zhizi in Chinese), *Glycyrrhizae Radix* et *Rhizome* (Gancao in Chinese), and *Menthae Haplocalycis Herba* (Bohe in Chinese), the auxiliary material is *Zingiberis Rhizoma Recens* (Shengjiang in Chinese) (National Pharmacopoeia Committee, 2020). Because of its composition, it is also called Danzhi Xiaoyao San in China. Due to its mood-regulating actions, it can be translated as free and easy wanderer plus (Wang et al., 2009). Because the preparation is initially in powder form, San can also be translated as powder (e.g. Jiawei Xiaoyao Powder). It was called Kami-Shoyo-San or Gami-Shoyo-San in Japan and South Korea, respectively (Gui, Fu, & Guo, 2015, Park et al., 2014, Shi, 2018, Yasui et al., 2009, Yue et al., 2019, Zhang et al., 2007). A variety of dosage forms are widely used in clinical in China, including mixtures, pills, granules, and capsules. There are powder and tablet forms in Kampo medicines in Japan, which are often used in Kampo hospitals (Guo et al., 2019). In *the Chinese Pharmacopoeia*, it is used as pills and mixtures. In other drug standards, there are granules, capsules, and tablets. In terms of efficacy, the therapeutic effects of these dosage forms are the same (Yue et al., 2019, Wang et al., 2015).

In most of the clinical research on TCM, JWXYS is not prescription preparation, but a prescription by a doctor. The dosage of the ten drugs in JWXYS is adjusted according to the patient’s symptoms. Some other Chinese medicines may be added according to other symptoms of patients. This is in line with the characteristic of TCM: syndrome differentiation and treatment, similar to the concept of precision medicine in modern medicine, which comprehensively considers the patient's condition, physical condition, living environment, and other factors to provide personalized treatment to the patient. In this review, some of the studies followed the concept of syndrome differentiation and treatment and other herbal medicines to the ten herbal medicines, and we will highlight these studies in subsequent articles.

This review summarized the existing research on JWXYS in the treatment of anxiety disorder and anxiety which aims to deepen the understanding of the therapeutic effects of JWXYS.

## 2 History and clinical application of JWXYS

In TCM, JWXYS has the effects of soothing the liver and clearing away heat, removing the stagnancy of nutritive *Qi*. So, it can be used to treat a variety of related diseases or alleviate a variety of related symptoms such as liver and spleen blood deficiency, smothery and night sweats, abdominal pain, vertigo, blurred vision, stun and uneasiness, cheeks and mouth dryness, irregular menstruation, fever, and other symptoms. At present, JWXYS was mainly used to treat anxiety (Lu, Shi, Chen, & Wei, 2020), depression (Guo, 2018), insomnia, and other mental illnesses. Moreover, JWXYS can also treat gynecology (Kimura et al., 2007), stomach disease and internal heat, cough, diabetes (Liu & Dai, 2020) and liver cancer (Tsai et al., 2019). Table 1 showed the specific history and clinical applications by successive generations of healers.

**Table 1:** Specific history and clinical application of JWXYS.

Publications	Published year	TCM applications	Corresponding diseases
*Internal Medicine Abstract*	1529	Deficiency of liver, spleen and blood; night sweating and headache; dizziness and anxiety; menstrual irregularities	Fever; anxiety disorder; diabetes; irregular menstruation
*Certificate and Rule Standards-Women's Section*	1602	Postpartum fever; dry mouth and thirst; cracked lips and sores	Postpartum depression; fever
*Six Books on Medical Strategy*	1903	Deficiency of blood and excessive heat; deficient in *yin* and prosperous in wood	Amenorrhea; sweating profusely
*Theory of The Origin of Various Diseases*	1773	Accumulation heat in the spleen; phlegm and saliva trapped in blood	Hemoptysis.
*Treatment of Female Diseases*	1724	Depression, anger and liver injury; chills, fever, dreadful pain and distress	Bipolar disorder; anxiety disorder.
*Book of Syndrome Differentiation and Treatment*	1687	Anger and rage; reversal of liver *qi*; excessive heat; deficiency of blood and *qi*	Bipolar disorder; fever; otitis media; premenstrual syndrome.

## 3 Pre-clinical studies

### 3.1 Animal behavior studies

JWXYS of various formulations exhibited an anxiolytic effect in experimental animal models of anxiety. The retrieved animal behavior experiment results are shown in Table 2. JWXYS significantly reduced anxiety-like behaviors in experimental animal models in animal behavior experiments. In Elevated plus maze (EPM), the number and time of entering the open arm of the animal taking JWXYS increased; in the open field test (OFT), the moving distance of the animal increased, and the number and time of entering the central area increased; in tail suspension test (TST) and forced swimming test (FST), the immobility time decreased; and in sucrose preference test, the intake of sugar water increased.

**Table 2:** Animal behavioral test.

Animals	Treatments	Composition of JWXYS	Dosages of JWXYS	Behavioral tests	Findings	References
SD rats (female)	Chronic stress	No detailed explanation	5.256 g/kg, 52.56 g/kg	EMP	TO%, EO%↑	(Cao, Gui, Fu, Guo, & Fu, 2016)
SD rats (male)	Immobilization stress	ASR 4 g, PRA 4 g, *Poria* 4 g, AMR 4 g, BR 4 g, LT 4 g, GRR 2 g, MHH 2 g, ZRR 6 g	0.67 g/kg, 1.34 g/kg	FST	Immobility time↓	(Park et al., 2007)
Wistar rats (male)	CUMS	No detailed explanation	Petroleum ether soluble fraction: 0.086 and 0.172 g/kg; Water-EtOH soluble fraction: 1.804 and 3.608 g/kg	SPT; FST; TST; OFT	Sucrose preferences↑ Duration of immobility↓ Duration of immobility↓ Locomotor activity and the rearing counts↑	(Wu et al., 2016)
SD rats (male)	CUMS	No detailed explanation	1.2 g/kg	SPT; OFT; FST	Sucrose preferences↑ Locomotor activity and the rearing counts↑ Duration of immobility↓	(Zhu et al., 2020)
ICR mice (male) SD Rats (male)	/	No detailed explanation	2.5 mg/kg, 5 mg/kg, 10 mg/kg	NSF; Vogel-type conflict test; EPM	Latency to feed↓ Food consumption (-) Number of shocks↑ TO%, EO%↑	(Qiu et al., 2015)
Wistar rats (male)	Predators exposed & Vogel's drinking conflict test	No detailed explanation	10.5 g/kg	EPM	TO%, EO%↑	(Song, Tang, Zhao, & Wang, 2014)
Wistar rats (male)	Predators exposed & Vogel's drinking conflict test	No detailed explanation	10.5 g/kg	OFT; EPM	Locomotor activity and the rearing counts↑ TO%, EO%↑	(Wang et al., 2014)

Note: CUMS: Chronic unpredictable mild stress; EMP: Elevated plus maze; FST: Forced swimming test; NSF: Novelty suppressed feeding; SPT: Sucrose preference test; TST: Tail suspension test; OFT: Open field test. AMR: *Atractylodis Macrocephalae Rhizoma*; ASR: *Angelicae Sinensis Radix*; BR: *Bupleuri Radix*; GRR: *Glycyrrhizae Radix* et *Rhizome*; LT: *Liriopis tuber*; MHH: *Menthae Haplocalycis Herba*; PRA: *Paeoniae Radix Alba*; ZRR: *Zingiberis Rhizoma Recens*.

### 3.2 Therapuetical mechanism of JWXYS for anxiety disorders

#### 3.2.1 Central nervous system and hypothalamic–pituitaryadrenal (HPA) axis

The mechanism research of JWXYS mainly focused on the central nervous system, especially the hippocampus, cortex, and amygdala. In addition, the HPA axis has also been the main focus of researchers. HPA axis disorders are common in animal models of anxiety, and JWXYS can normalize the HPA axis by decreasing the plasma levels of corticosterone (CORT), corticotropin-releasing hormone (CRH), and adrenocorticotropic hormone (ACTH) and increasing arginine vasopressin (AVP) levels in animals (Cao, Gui, Fu, Guo, & Fu, 2016, Wu et al., 2016). CRH, ACTH, and AVP in the hypothalamus of animal models also returned to normal after the administration of JWXYS. The plasma, cortical and hypothalamus responses of the corresponding hormones to the high dose of JWXYS were similar to those of the positive drug imipramine, suggesting that it is more effective in treating symptoms of HPA axis disorders in anxiety disorders (Wu et al., 2016).

The anxiolytic and neuroprotective effects of JWXYS may also be achieved by attenuating chronic stress-induced up-regulation of *α*-synuclein and stress-induced downregulation of mRNA and protein levels of protein phosphatase 2A (PP2A) in the hippocampal region of the brain. High doses of JWXYS were able to significantly down-regulate *α*-synuclein and up-regulate PP2A levels, restoring neuronal function (Cao, Gui, Fu, Guo, & Fu, 2016). *In vitro* cell experiments also demonstrated that senkyunolide A (SenA), one of the main components of JWXYS, protects against CORT-induced apoptosis in PC12 cells. SenA increased the CORT-induced decrease in PP2A activity while decreasing the expression of p-PP2A, *α*-synuclein, and p*-α*-synuclein (Ser129). The neuroprotection of neurons also depends on inhibition of *α*-synuclein function, and regulation of *α*-synuclein function will affect PP2A activity in a negative-loop manner (Gong, Zhang, Guo, & Fu, 2018). Besides, the administration of JWXYS (5 and 10 mg/kg, p.o.) increased allopregnanolone levels in the prefrontal cortex and hippocampus (Qiu et al., 2015). Reduced levels of allopregnanolone in the peripheral blood or cerebrospinal fluid were found to be associated with major depression, anxiety disorders, premenstrual dysphoric disorder, negative symptoms in schizophrenia, or impulsive aggression. The importance of allopregnanolone for the regulation of emotion and its therapeutic use in depression and anxiety may not only involve GABAergic mechanisms, but probably also includes enhancement of neurogenesis, myelination, neuroprotection, and regulatory effects on HPA axis function (Guo et al., 2019).

#### 3.2.2 Regulation of neurotransmitter levels

In addition to the effect on hormones, JWXYS can also increase the monoamine neurotransmitters in the hippocampus, which was shown by the increase of 5-hydroxytryptamine (5-HT) and dopamine (DA) content, but the content of 5-hydroxyindoleacetic acid (5-HIAA) does not change (Cheng, Wen, Sun, Leng, & Xia, 2020, Wu et al., 2016), indicating that JWXYS may increase 5-HT synthesize and reduce its metabolism and affect the content of 5-HT. The amino acid neurotransmitters in the hippocampus are also affected by JWXYS. The increase in glutamate content in the hippocampus of rats caused by Chronic unpredictable mild stress (CUMS) can be reversed by large doses of JWXYS, but *γ*-aminobutyric acid (GABA) is not affected (Wu et al., 2016).

#### 3.2.3 Increasing hippocampal neurogenesis

JWXYS also improves anxiety by increasing hippocampal neurogenesis (Park et al., 2007). The neurons in the hippocampus and amygdala of normal rats are large and round, and the cells are arranged in an orderly and concentrated manner. Many neurons in the anxiety model rats have undergone nuclear division, excessive discoloration and deformation of the cells, and loose irregular cell arrangements. However, the nerve cells in the hippocampus and amygdala of rats treated with JWXYS improved significantly, and the cell layout was relatively orderly (Ma, Tang, Zhao, Dong, & Wang, 2015). The hippocampal dentate gyrus slices of rats with 5-Bromo-2′-deoxyuridine single labeling (BrdU^+^) and BrdU/neuron nuclear antibody double labeling (BrdU^+^/NeuN^+^) showed that the number of BrdU^+^ cells in anxiety model rats increased, while the JWXYS treated rat showed a significant increase in the number of BrdU^+^ and BrdU^+^/NeuN^+^ cells. This indicates that JWXYS can promote the proliferation of neural stem cells in the dentate gyrus (DG) area of generalized anxiety disorder (GAD) rats and the directed differentiation into neurons, enhance the body's nerve repair and regeneration capabilities, and thereby improve clinical symptoms (Dong et al., 2015).

## 4 Clinical studies

The clinical trial results are shown in Table 3. Studies have shown that JWXYS has a therapeutic effect on patients with anxiety disorder and anxiety. Using Hamilton Anxiety Scale (HAMA), Self-Rating Anxiety Scale (SAS), and other scales to evaluate the anxiety level of patients, researchers found that oral administration of JWXYS alone can reduce the anxiety level of patients (Park et al., 2014, Yasui et al., 2009). The combined use of JWXYS and positive drugs with definite efficacy can relieve symptoms while reducing adverse reactions (Jin, 2014, Xiong & Song, 2018, Yue et al., 2019). Other scales that can evaluate the symptoms of anxiety patients were also used by researchers. After treatment with JWXYS, the patient’s sleep time and quality were significantly improved (Li, 2019), quality of life improved (Xiong & Song, 2018), clinical symptoms reduced, and symptoms of TCM symptoms improved (Li, 2019, Park et al., 2014, Wang, 2014). In one study, the serum IL-6 levels of patients treated with JWXYS also decreased, but other cytokines remained unchanged (Yasui et al., 2009).

**Table 3:** Clinical studies of JWXYS.

Patients	Drugs	Composition of JWXYS	Positive drugs	Methods and findings	References
147 patients with GAD	JWXYS individual extract mixture or multi-compound extract	ASR 1 g, PRA 1 g, *Poria* 1 g, AMR 1 g, BR 1 g, MC 0.67 g, GF 0.67 g, GRR 0.67 g, ZRR 0.33 g, MHH 0.33 g	Placebo	HAMA: Multi-compound extract group < Individual extract mixture group and placebo group; WHOQOL-BREF: Multi-compound extract group > Individual extract mixture group and placebo group; K-BDI: Multi-compound extract group < Individual extract mixture group	(Park et al., 2014)
76 women with anxiety and mild depression as menopausal symptoms	JWXYS	ASR 3 g, PRA 3 g, *Poria* 3 g, AMR 3 g, BR 3 g; MC 2 g, GF 2 g, GRR 1.5 g, ZRR 1 g, MHH 1 g	Paroxetine	Greene's climacteric scale: Paroxetine group > JWXYS group; Serum cytokines: IL-6↑	(Yasui et al., 2009)
256 patients with GAD	JWXYS combined with acupuncture	PRA 15 g, *Poria* 15 g, AMR 15 g, BR 15 g, MC 15 g, GF 15 g, MHH 15 g, CSF 15 g; ASR 9 g, ZRR 9 g, GRR 3 g	Chemical medicine	SAS and HAMA: JWXYS combined with acupuncture < Chemical medicine	(Liu, 2020)
60 anxiety patients with liver stagnation and fire-type insomnia	JWXYS combined with zopiclone	ASR 15 g, PRA 15 g, *Poria* 15 g, AMR15 g, BR 15 g, AC 15 g, PMC 15 g, CR 15 g, MC 12 g, GF12 g, GRR 3 g, FF 20 g, OC 20 g	Zopiclone	PSQI, HAMA and TCM syndrome scale: JWXYS combined with zopiclone < Zopiclone	(Li, 2019)
80 cases of GAD of pathogenic fire derived from the stagnation of liver-qi type	JWXYS	ASR 10 g, PRA 10 g, *Poria* 10 g, AMR 10 g, GF 10 g, BR 5 g, MC 8 g; GRR 5 g	Flumethiazide Medrotixin	HAMA and SAS: JWXYS group < Flumethiazide medrotixin group.	(Shi, 2018)
200 patients with anxiety	JWXYS combined with anxiolytic drugs	ASR 15 g, PRA 15 g, *Poria* 15 g, AMR 12 g, BR 15 g, MC 15 g, GF15 g, GRR 6 g, MHH 6 g, FF 30 g, OC 30 g, PMC 30 g	Anxiolytic drugs	HAMA: JWXYS combined with anxiolytic drugs < anxiolytic drugs (*P* < 0.05); Sleep time scores: JWXYS combined with anxiolytic drugs < anxiolytic drugs (*P* < 0.05); Life quality: Observation group > Control group (*P* < 0.05); Adverse reactions: No statistically significant difference (*P* > 0.05).	(Xiong & Song, 2018)
75 perimenopausal women with emotional disorders	JWXYS	ASR, PRA, *Poria*, AMR, BR, MC, GF, GRR, MHH, ZRR	Paroxetine	HAMA and HAMD: Both groups improved (*P* < 0.01), and no statistical difference between the two groups (*P* > 0.05); TESS: Different dosage forms of JWXYS < Paroxetine (*P*＜0.01).	(Yue et al., 2019)
46 cases of climacteric syndrome patients with emotional symptoms	JWXYS + diethylstilbestrol, gamma-oryzanol and vitamin	ASR 10 g, PRA 10 g, *Poria* 10 g, AMR 10 g, BR 10 g, MC 10 g, GF 10 g, GRR 6 g, MHH 6 g, ZRR 6 g	Diethylstilbestro, gamma-oryzanol and vitamin	SAS and SDS: JWXYS combined with diethylstilbestrol, gamma-oryzanol and vitamin < diethylstilbestrol, gamma-oryzanol and vitamin (*P* < 0.05); Clinical symptoms: Total effective rate improved (*P* < 0.05); Side effects: No statistically significant difference (*P* > 0.05).	(Jin, 2014)
104 cases of gastric ulcer patients with anxiety	Weikang capsule combined with JWXYS	ASR, PRA, *Poria*, AMR, BR, MC, GF, GRR, MHH, ZRR	Ranitidine capsules	SAS: Weikang capsule combined with JWXYS < Ranitidine capsules (*P* < 0.05).	(Wang, 2014)

GAD: Generalized anxiety disorder; HAMA: Hamilton Anxiety Scale; HAMD: Hamilton Depression Scale; WHOQOL-BREF: World Health Organization Quality of Life Instruments; K-BDI: Korean Beck Depression Inventory; SAS: Self-Rating Anxiety Scale; PSQI: Pittsburgh sleep quality index; TESS: Treatment-Emergent-Symptom Scale. AC: *Albiziae Cortex*; AMR: *Atractylodis Macrocephalae Rhizoma*; ASR: *Angelicae Sinensis Radix*; BR: *Bupleuri Radix*; CR: *Curcumae Radix*; CSF: *Citri Sarcodactylis Fructus*; FF: *Fossil Fragments*; GF: *Gardeniae Fructus*; GRR: *Glycyrrhizae Radix* et *Rhizome*; LT: *Liriopis tuber*; MC: *Moutan Cortex*; MHH: *Menthae Haplocalycis Herba*; OC: *Ostreae Concha*; PMC: *Polygoni Multiflori Caulis*; PRA: *Paeoniae Radix Alba*; ZRR: *Zingiberis Rhizoma Recens*.

## 5 Discussion

**Fig. 2:**
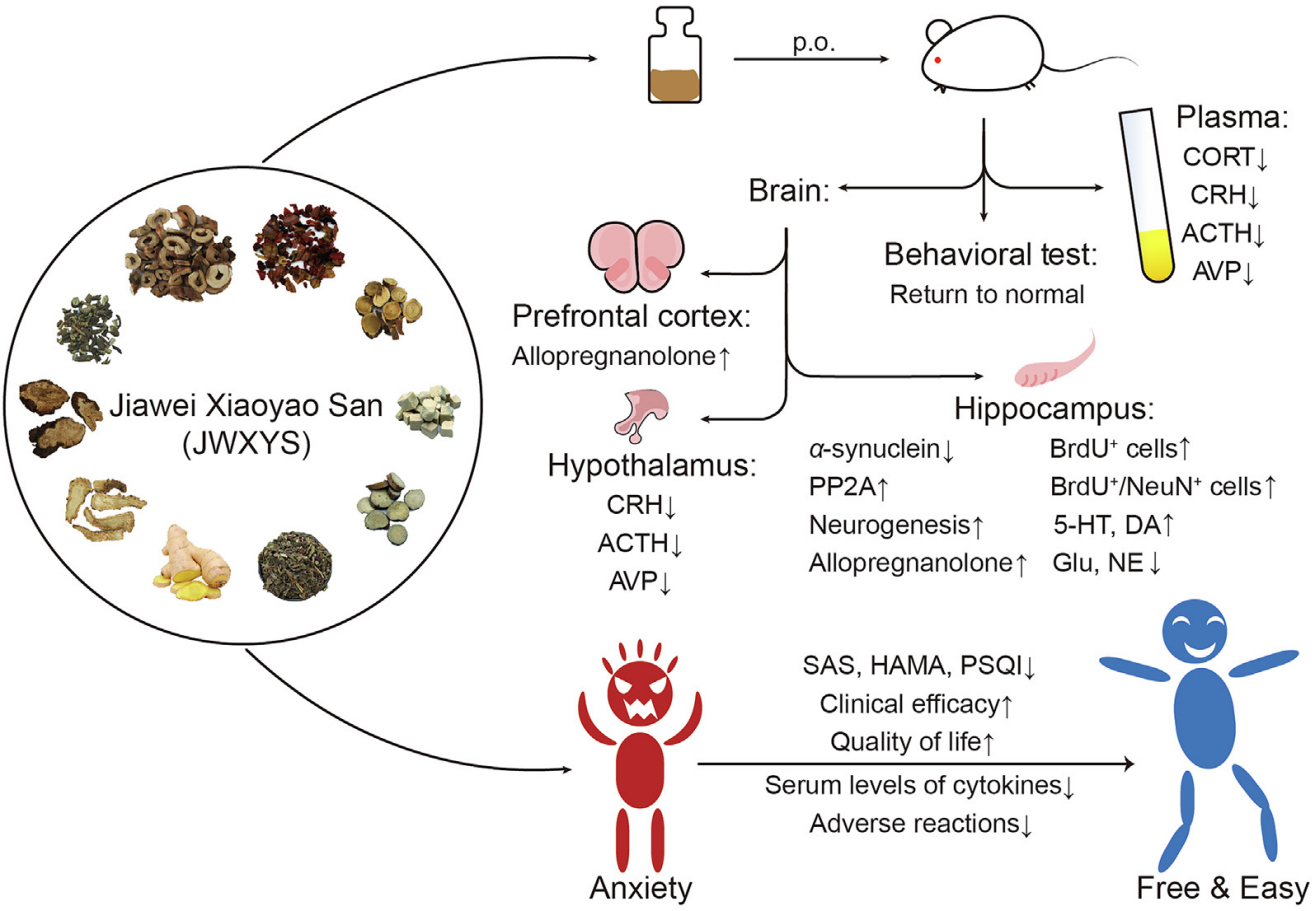
A brief anxiolytic mechanism of JWXYS. CORT: Corticosterone; CRH: Corticotropin-releasing hormone; ACTH: Adrenocorticotropic hormone; AVP: Arginine vasopressin; PP2A: Protein phosphatase 2A; BrdU^+^: 5-Bromo-2′-deoxyuridine single labeling; BrdU^+^/NeuN^+^: BrdU/neuron nuclear antibody double labeling; HAMA: Hamilton anxiety scale; SAS: Self-rating anxiety scale; PSQI: Pittsburgh sleep quality index.

So far, there have been some studies about the clinical efficacy and mechanism of JWXYS (Fig. 2). However, the number of samples used in these clinical studies and the diagnostic criteria were different which may be because there is no exact pathogenesis for anxiety disorders. The scales are usually used in the diagnosis of anxiety disorders, among which HAMA is the most used and important diagnostic scale for anxiety disorders, but it cannot well measure the anxiety state caused by various other diseases. At the same time, HAMA and Hamilton Depression Scale (HAMD) have overlaps in some items, such as physical anxiety, depressed mood and gastrointestinal symptoms. Therefore, HAMA cannot distinguish anxiety from depression. Other commonly used scales, such as SAS, Pittsburgh sleep quality index (PSQI), etc. are also used in clinical research to further evaluate patients' anxiety and other symptoms.

In the existing research, JWXYS has a certain anxiolytic effect in the anxiety-like animal. The mechanism of JWXYS is mainly to improve the levels of the HPA axis and other hormones, regulate the content of related enzymes and neurotransmitters in the brain and affect the nerves of the hippocampus. However, according to the current literature, the pathogenesis and treatment options of anxiety disorders are not limited to the central nervous system. Peripheral circulation and the microbe-gut-brain axis are research hotspots in recent years. Compared with chemical drugs, research on the mechanism of Chinese herbal compound prescriptions should pay more attention to these new ideas. For example, the effects of Xiaoyaosan, the predecessor of JWXYS, on the gut microbiota of depressed rats have been studied. This study shows that Xiaoyaosan can improve the gut microbiota disorder caused by CUMS and relieve the depressive state of rats (Zhu et al., 2019). JWXYS may have a similar effect, which needs further research.

The idea of syndrome differentiation and treatment in TCM makes the prescription of JWXYS in TCM clinics not fixed. Except for the ten kinds of drugs, which will not change, other TCMs added according to the patient's symptoms are uncertain. Also, the dosages of various TCMs were also different. To a certain extent, this can be understood as precision medicine. Each Chinese medicine is regarded as a medicine, and different medicines are used together. But unlike precision medicine, traditional Chinese medicine doctors compatibilized medicines based on traditional Chinese medicine concepts.

In clinical studies, we have seen only one experiment to detect serum cytokines in patients (Yasui et al., 2009), which is rare in the research of other diseases. However, when JWXYS is used to treat other diseases such as hepatitis, various biochemical indicators of the patient will be tested as the basis for diagnosis and evaluation of treatment effects (Ling, Mao, Chen, Liang, & Wang, 2017, Chien et al., 2014). Besides, smoking and drinking (Fluharty, Taylor, Grabski, & Munafò, 2017, Serrano et al., 2018) and other bad lifestyle habits are also related to the occurrence of anxiety. Quitting smoking (Zvolensky et al., 2018) and quitting alcohol (Gupta & Sharma, 2019) can promote the development of anxiety. In the process of taking Chinese medicine, smoking and drinking are usually not allowed, which will also have a certain impact on the treatment process. However, to consider the current research, an in-depth research is needed in the future.

JWXYS is a popular medicine in East Asia. It is often used to treat anxiety and has been widely recognized in East Asia. However, due to differences in language and medical systems, most of the research articles on JWXYS are in Chinese, Japanese and Korean, and only some studies have been published in international academic journals. Although the English literature and authoritative Chinese literature on JWXYS were reviewed, some research articles in Japanese or Korean may be missed. In addition, in clinical studies, the observation group was often given JWXYS and another positive drug with clear efficacy and the control group was given the positive drug alone, which led to our inability to distinguish whether the symptoms of the observation group were improved by JWXYS. Besides, due to the idea of ‘syndrome differentiation and treatment’ in Chinese medicine, the composition of JWXYS is not fixed, which is very common in Chinese medicine, because the purpose of Chinese medicine is to cure patients, not to consider whether this formula has a prescription. In summary, to comprehensively understand the therapeutic effects and mechanism of JWXYS, more clinical trials and mechanism studies with controllable conditions are needed.

## 6 Conclusion

As an ancient Chinese medicine formula, clinical studies have shown that JWXYS has a certain effect in the treatment of anxiety and anxiety disorders, and at the same time, it can reduce the adverse reactions of patients It is a potential anxiolytic drug at a cheap price. During treatment, JWXYS can be considered a supplementary treatment. However, the mechanism of action of JWXYS is still unclear and needs further study.

## Authors’ contributions

Jiaqi Xie wrote the manuscript, Dandan Xu participate in literature collection and sorting, Can Wang and Jianmei Huang proofread and edited the manuscript. All authors reviewed and approved the manuscript.

## Declaration of Competing Interest

The authors declare that they have no known competing financial interests or personal relationships that could have appeared to influence the work reported in this paper.

## References

[b0005] American Psychiatric Association (2015).

[b0010] Baldwin D., Woods R., Lawson R., Taylor D. (2011). Efficacy of drug treatments for generalised anxiety disorder: Systematic review and meta-analysis. British Medical Journal.

[b0015] Bandelow B., Michaelis S., Wedekind D. (2017). Treatment of anxiety disorders. Dialogues in Clinical Neuroscience.

[b0020] Cao G.P., Gui D., Fu L.D., Guo Z.K., Fu W.J. (2016). Anxiolytic and neuroprotective effects of the Traditional Chinese Medicinal formulation Dan-zhi-xiao-yao-san in a rat model of chronic stress. Molecular Medicine Reports.

[b0025] Celano C.M., Millstein R.A., Bedoya C.A., Healy B.C., Roest A.M., Huffman J.C. (2015). Association between anxiety and mortality in patients with coronary artery disease: A meta-analysis. American Heart Journal.

[b0030] Cheng J.L., Wen X.Y., Sun Y.H., Leng J., Xia M. (2020). The effects of Jiawei Xiaoyao Powder on the levels of dopamine and serotonin in hepatocellular carcinoma rats. Chinese Journal of Neuroanatomy.

[b0035] Chien, S. C., Chang, W. C., Lin, P. H., Chang, W. P., Hsu, S. C., Chang, J. C., Wu, Y. C., Pei, J. K., & Lin, C. H. (2014). A Chinese herbal medicine, jia-wei-xiao-yao-san, prevents dimethylnitrosamine-induced hepatic fibrosis in rats. *The Scientific World Journal*, 2014, 217525. 10.1155/2014/217525 PMC4065731 24995353

[b0040] Dong N., Zhao R.Z., Xu S., Wang W.L., Li D.M., Tang Q.S. (2015). Intervention effects of Danzhi Xiaoyao Power on proliferation and differentiation of neural stem cell in dentate gyms in rats with generalized anxiety disease. Beijing Journal of Traditional Chinese Medicine.

[b0045] Ferrari A.J., Somerville A.J., Baxter A.J., Norman R., Patten S.B., Vos T., Whiteford H.A. (2013). Global variation in the prevalence and incidence of major depressive disorder: A systematic review of the epidemiological literature. Psychological Medicine.

[b0050] Fluharty M., Taylor A.E., Grabski M., Munafò M.R. (2017). The association of cigarette smoking with depression and anxiety: A systematic review. Nicotine & Tobacco Research: Official Journal of the Society for Research on Nicotine and Tobacco.

[b0055] Gong S.L., Zhang J., Guo Z.K., Fu W.J. (2018). Senkyunolide A protects neural cells against corticosterone-induced apoptosis by modulating protein phosphatase 2A and *α*-synuclein signaling. Drug Design, Development and Therapy.

[b0060] Gui D., Fu W.J., Guo Z.K. (2015). Advances in mechanisms of molecular biology of Danzhi Xiaoyao San in anti-hippocampal neuronal damage of anxiety disorder. Chinese Journal of Experimental Traditional Medical Formulae.

[b0065] Guo J.H. (2018). Analysis Jiawei Xiaoyao Powder clinical treatment effect to patients with depression. World Journal of Sleep Medicine.

[b0070] Guo Q.Y., Ebihara K., Shimodaira T., Fujiwara H., Toume K., Dibwe D.F., Awale S., Araki R., Yabe T., Matsumoto K. (2019). Kami-shoyo-san improves ASD-like behaviors caused by decreasing allopregnanolone biosynthesis in an SKF mouse model of autism. PLoS One.

[b0075] Gupta G.L., Sharma L. (2019). Bacopa monnieri abrogates alcohol abstinence-induced anxiety-like behavior by regulating biochemical and *Gabra1*, *Gabra4*, *Gabra5* gene expression of GABA(A) receptor signaling pathway in rats. Biomedicine & Pharmacotherapy.

[b0080] Jin M.Q. (2014). Clinical observation of Danzhixiaoyao Powder combined with Western medicine in treatment of climacteric syndrome with emotional symptoms. Journal of Hubei University of Chinese Medicine.

[b0085] Kalin N.H. (2020). Novel insights into pathological anxiety and anxiety-related disorders. American Journal of Psychiatry.

[b0090] Kasteenpohja T., Marttunen M., Aalto-Setälä T., Perälä J., Saarni S.I., Suvisaari J. (2018). Outcome of depressive and anxiety disorders among young adults: Results from the Longitudinal Finnish Health 2011 Study. Nordic Journal of Psychiatry.

[b0095] Kessler R.C., Amminger G.P., Aguilar-Gaxiola S., Alonso J., Lee S., Ustün T.B. (2007). Age of onset of mental disorders: A review of recent literature. Current Opinion in Psychiatry.

[b0100] Kimura Y., Takamatsu K., Fujii A., Suzuki M., Chikada N., Tanada R., Kume Y., Sato H. (2007). Kampo therapy for premenstrual syndrome: Efficacy of Kamishoyosan quantified using the second derivative of the fingertip photoplethysmogram. Journal of Obstetrics and Gynaecology Research.

[b0105] Konnopka A., König H. (2020). Economic burden of anxiety disorders: A systematic review and Meta-analysis. Pharmacoeconomics.

[b0110] Li J. (2019).

[b0115] Ling C.P., Mao D.W., Chen Y.Q., Liang X.Y., Wang T.S. (2017). Clinical study on efficacy of modified Xiaoyaosan for patients with chronic hepatitis B in syndrome of liver stagnation and spleen deficiency and blood stasis. Liaoning Journal of Traditional Chinese Medicine.

[b0120] Liu W.J., Dai X.J. (2020). Clinical observation of modified Danzhi Xiaoyao Powder combined with estazolam in treating diabetes complicated with insomnia. Diabetes New World.

[b0125] Liu Y.H. (2020). Clinical analysis on Danzhi Xiaoyao Powder combined with acupuncture in the treatment of generalized anxiety disorder. Chinese Medicine Modern Distance Education of China.

[b0130] Lu D.C., Shi A.H., Chen S., Wei S.S. (2020). Research progress of Dan Xiaoyao San in treating anxiety. Chinese Journal of Ethnomedicine and Ethnopharmacy.

[b0135] Ma X.F., Tang Q.S., Zhao R.Z., Dong N., Wang R. (2015). Effects of Danzhi Xiaoyao San on morphological changes of encephalic regions in the anxiety model rats. Journal of Beijing University of Traditional Chinese Medicine.

[b0140] Merikangas K.R., He J.P., Burstein M., Swanson S.A., Avenevoli S., Cui L., Benjet C., Swendsen K., Georgiades J. (2010). Lifetime prevalence of mental disorders in U.S. adolescents: Results from the National Comorbidity Survey Replication-Adolescent Supplement (NCS-A). Journal of the American Academy of Child and Adolescent Psychiatry.

[b0145] National Pharmacopoeia Committee (2020). National Pharmacopoeia Committee.

[b0150] Noma H., Cipriani A., Cuijpers P., Furukawa T., Vinkers C., Karyotaki E. (2019). A network meta-analysis of the effects of psychotherapies, pharmacotherapies and their combination in the treatment of adult depression. World Psychiatry.

[b0155] Park D.M., Kim S.H., Park Y.C., Kang W.C., Lee S.R., Jung I.C. (2014). The comparative clinical study of efficacy of Gamisoyo-San (Jiaweixiaoyaosan) on generalized anxiety disorder according to differently manufactured preparations: Multicenter, randomized, double blind, placebo controlled trial. Journal of Ethnopharmacology.

[b0160] Park S.W., Kim Y.K., Lee J.G., Kim S.H., Kim J.M., Yoon J.S., Park Y.K., Kim Y.K., Lee Y.H. (2007). Antidepressant-like effects of the traditional Chinese medicine Kami-shoyo-san in rats. Psychiatry and Clinical Neurosciences.

[b0165] Qiu Z.K., He J.L., Liu X., Lai S., Ma J.C., Zeng J., Li Y., Wu H.W., Chen Y., Shen Y.G., Chen J.S., Luo M. (2015). The role of allopregnanolone in the anxiolytic-like effect of free and easy wanderer plus (FEWP), a polyherbal preparation. Neuroscience Letters.

[b0170] Rotvig C., Christensen A.V., Juel K., Svendsen J.H., Jørgensen M.B., Rasmussen T.B., Berg S.K. (2022). The association between cardiac drug therapy and anxiety among cardiac patients: Results from the national DenHeart survey. BMC Cardiovascular Disorders.

[b0175] Roy-byrne P.P., Cowley D.S. (1994). Course and outcome in panic disorder: A review of recent follow-up studies. Anxiety..

[b0180] Roy-Byrne P.P., Craske M.G., Stein M.B. (2006). Panic disorder. Lancet.

[b0185] Ruscio A.M., Hallion L.S., Lim C.C.W., Aguilar-Gaxiola S., Al-Hamzawi A., Alonso J., Andrade L.H., Borges G., Bromet E.J., Bunting B., Caldas de Almeida J.M., Demyttenaere K., Florescu S., de Girolamo G., Gureje O., Haro J.M., He Y., Hinkov H., Hu C., de Jonge P., Karam E.G., Lee S., Lepine J.P., Levinson D., Mneimneh Z., Navarro-Mateu F., Posada-Villa J., Slade T., Stein D.J., Torres Y., Uda H., Wojtyniak B., Kessler R.C., Chatterji S., Scott K.M. (2017). Cross-sectional comparison of the epidemiology of DSM-5 generalized anxiety disorder across the globe. JAMA Psychiatry.

[b0190] Serrano A., Pavon F.J., Buczynski M.W., Schlosburg J., Natividad L.A., Polis I.Y., Stouffer D.G., Zorrilla E.P., Roberto M., Cravatt B.F., Martin-Fardon R., Rodriguez de Fonseca F., Parsons L.H. (2018). Deficient endocannabinoid signaling in the central amygdala contributes to alcohol dependence-related anxiety-like behavior and excessive alcohol intake. Neuropsychopharmacology: Official Publication of the American College of Neuropsychopharmacology.

[b0195] Shi D. (2018). Clinical study on Danzhi Xiaoyao Powder in the treatment of generalized anxiety (disorder) of pathogenic fire derived from stagnation of liver-qi type. Chinese Medicine Modern Distance Education of China.

[b0200] Slee A., Nazareth I., Bondaronek P., Liu Y., Cheng Z., Freemantle N. (2019). Pharmacological treatments for generalised anxiety disorder: A systematic review and network meta-analysis. The Lancet.

[b0205] Song C.L., Tang Q.S., Zhao R.Z., Wang W.L. (2014). Effects of Danzhi Xiaoyao Powder on ethological changes in model rats with exposure to predators and conflict of drinking water. Beijing Journal of Traditional Chinese Medicine.

[b0210] Tiller J.W.G. (2013). Depression and anxiety. The Medical Journal of Australia.

[b0215] Tsai, F. J., Cheng, C. F., Chen, C. J., Lin, C. Y., Wu, Y. F., Li, T. M., Chuang, P. H., Wu, Y. C., Lai, C. H., Liu, X., Tsang, H., Lin, T. H., Liao, C. C., Huang, S. M., Li, J. P., Lin, J. C., Lin, C. C., Liang, W. M., & Lin, Y. J. (2019). Effects of Chinese herbal medicine therapy on survival and hepatic outcomes in patients with hepatitis C virus infection in Taiwan. *Phytomedicine*, 57, 30–38. 10.1016/j.phymed.2018.09.237 30668320

[b0220] Wang H.N., Peng Y., Tan Q.R., Wang H.H., Chen Y.C., Zhang R.G., Wang Z.Z., Guo L., Zhang Y., Liu Z.J. (2009). Free and Easy Wanderer Plus (FEWP), a polyherbal preparation, ameliorates PTSD-like behavior and cognitive impairments in stressed rats. Progress in Neuro-Psychopharmacology & Biological Psychiatry.

[b0225] Wang W.H., Yue L.F., Du M.S., Gao Y.M., Wang Q., Zhang H., Liu S.Z. (2015). Evaluation on difference of therapeutic efficacy of Jiawei Xiaoyao Granules and Pills in treatment of emotional disorder during perimenopause based on Greene Climacteric Scale. China Journal of Traditional Chinese Medicine and Pharmacy.

[b0230] Wang W.L., Tang Q.S., Zhao R.Z., Xu S., Yang X.K., Huang X. (2014). Effect of Danzhi Xiaoyao San on ethology of rat model in Vogel’s drinking conflict test and predator exposing test. Journal of Beijing University of Traditional Chinese Medicine.

[b0235] Wang X.X. (2014). Curative observation of using Weikang Capsule combines with Jiawei Xiaoyao Powder to treat gastric ulcer with anxiety. Journal of Sichuan of Traditional Chinese Medicine.

[b0240] Wu L.L., Liu Y., Yan C., Pan Y., Su J.F., Wu W.K. (2016). Antidepressant-like effects of fractions prepared from Danzhi-Xiaoyao-San Decoction in rats with chronic unpredictable mild stress: Effects on hypothalamic-pituitary-adrenal axis, arginine vasopressin, and neurotransmitters. Evidence-Based Complementary and Alternative Medicine.

[b0245] Xiong F., Song P.R. (2018). Observation on clinical effect of addition and subtraction therapy with Danzhi Xiaoyao Powder on the treatment of anxiety. Chinese Journal of Rational Drug Use.

[b0250] Yasui T., Yamada M., Uemura H., Ueno S.I., Numata S., Ohmori T., Tsuchiya N., Noguchi M., Yuzurihara M., Kase Y., & Irahara M. (2009). Changes in circulating cytokine levels in midlife women with psychological symptoms with selective serotonin reuptake inhibitor and Japanese traditional medicine. Maturitas.

[b0255] Yue L.F., Liu J., Wang W.H., Gao Y.Y., Zhang H., Wang D.M., Li Z.G. (2019). Evaluation of the efficacy of Jiawei Xiaoyao Powder in regulating perimenopausal mood disorders based on HAMA and HAMD. China Journal of Traditional Chinese Medicine and Pharmacy.

[b0260] Zhang Z.J., Kang W.H., Tan Q.R., Li Q., Gao C.G., Zhang F.G., Wang H.H., Ma X.C., Chen C., Wang W., Guo L., Zhang Y.H., Yang X.B., & Yang G.D. (2007). Adjunctive herbal medicine with carbamazepine for bipolar disorders: A double-blind, randomized, placebo-controlled study. Journal of Psychiatric Research.

[b0265] Zhu H.Z., Liang Y.D., Ma Q.Y., Hao W.Z., Li X.J., Wu M.S., Deng L.J., Li Y.M., & Chen J.X. (2019). Xiaoyaosan improves depressive-like behavior in rats with chronic immobilization stress through modulation of the gut microbiota. Biomedicine & Pharmacotherapy.

[b0270] Zhu Y.L., Li S.L., Zhu C.Y., Wang W., Zuo W.F., Qiu X.J. (2020). Metabolomics analysis of the antidepressant prescription Danzhi Xiaoyao Powder in a rat model of chronic unpredictable mild stress (CUMS). Journal of Ethnopharmacology.

[b0275] Zvolensky M.J., Garey L., Allan N.P., Farris S.G., Raines A.M., Smits J.A.J., Kauffman B.Y., Manning K., & Schmidt N.B. (2018). Effects of anxiety sensitivity reduction on smoking abstinence: An analysis from a panic prevention program. Journal of Consulting and Clinical Psychology.

